# Knockout of *Atg5* delays the maturation and reduces the survival of adult-generated neurons in the hippocampus

**DOI:** 10.1038/cddis.2015.406

**Published:** 2016-03-03

**Authors:** Y Xi, J S Dhaliwal, M Ceizar, M Vaculik, K L Kumar, D C Lagace

**Affiliations:** 1 Department of Cellular and Molecular Medicine, University of Ottawa, Ottawa K1H 8M5, Ontario, Canada

## Abstract

Autophagy is an evolutionarily conserved lysosomal degradation pathway that plays important roles in cell maintenance, expansion and differentiation. Removal of genes essential for autophagy from embryonic neural stem and precursor cells reduces the survival and inhibits neuronal differentiation of adult-generated neurons. No study has modified autophagy within the adult precursor cells, leaving the cell-autonomous role of autophagy in adult neurogenesis unknown. Here we demonstrate that autophagic flux exists in the adult dividing progenitor cells and their progeny in the dentate gyrus. To investigate the role of autophagy in adult hippocampal neurogenesis, we genetically deleted *Autophagy-related gene 5* (*Atg5*) that reduced autophagic flux and the survival of the progeny of dividing progenitor cells. This significant reduction in survival of adult-generated neurons is accompanied by a delay in neuronal maturation, including a transient reduction in spine density in the absence of a change in differentiation. The delay in cell maturation and loss of progeny of the Atg5-null cells was not present in mice that lacked the essential pro-apoptotic protein Bax (Bcl-2-associated X protein), suggesting that Atg5-deficient cells die through a Bax-dependent mechanism. In addition, there was a loss of Atg5-null cells following exposure to running, suggesting that Atg5 is required for running-induced increases in neurogenesis. These findings highlight the cell-autonomous requirement of Atg5 in the survival of adult-generated neurons.

In the adult brain, neurogenesis allows for the continuous development of adult-generated neurons in response to physiological and pathological stimuli. The neural progenitor cells (NPCs) within the neurogenic niche of the subventricular zone (SVZ) and subgranular zone (SGZ) give rise to adult-generated neurons within the olfactory bulb and dentate, respectively.^1, 2, 3^ The ability of the NPCs to proliferate, differentiate and integrate into circuitry to modify behavior makes understanding these cells and the factors that regulate these processes critical to develop new therapies. This is especially important for a number of diseases such as neurodegenerative diseases including Parkinson's and Huntington's diseases that are associated with reduced adult neurogenesis, as well as regenerative medicine strategies for recovery after stroke.^4, 5, 6^

Two groups have found that *in vivo* macroautophagy (hereafter referred to as autophagy) can regulate adult neurogenesis by examining the effect of deleting autophagy-related genes (*Atgs*). Yazdankhah *et al.*^7^ found that Ambra1 and Beclin1 heterozygous embryonic knockout mice have less proliferating NPCs in the SVZ and an associated reduction in neurogenesis in the olfactory bulb. Wang *et al.*^8^ found that conditional removal of *FIP200* (focal adhesion kinase (FAK) family interacting protein of *M*_r_ 200 K, also known as ULK1, an Atg1 homologue-interacting protein) from embryonic NPCs progressively depletes the number of postnatal NPCs, as well as reduces neurogenesis and increases astrogenesis. In contrast in the embryo, Lv *et al.*^9^ showed that a specific knockdown of the *Autophagy-related gene 5* (*Atg5*) increases proliferation and inhibits neuronal differentiation of embryonic NPCs during cortical development. These data suggest that embryonic and adult NPCs are altered when autophagy-related genes are deleted in the embryo. However, it remains unknown whether autophagy, independent of effects in the embryo, is directly required for NPCs and their progeny in the adult.

Here we tested the functional role of autophagy specifically in the adult brain by removing *Atg5* from dividing NPCs. We found that autophagic flux occurs in adult NPCs and that removal of *Atg5* is associated with a reduction in autophagic flux. In addition, we find that Atg5-null cells have a significant reduction in survival, as well as a delay in neuronal maturation. The reduction in neurogenesis occurred in the absence of altering proliferation or cell lineage. Furthermore, removal of *Bax* (Bcl-2-associated X protein) restored neurogenesis in the absence of *Atg5*, implicating Bax functions downstream of Atg5 to regulate the survival of adult-generated neurons. Finally, we showed that Atg5-dependent signaling is required for running-induced increases in the survival of the adult developing NPCs.

## Results

### Autophagic flux in hippocampal NPCs and their progeny

Autophagy is a highly dynamic process that includes the induction, initiation and elongation of the isolation membrane, forming the autophagosome that is cleared after fusion with the lysosome. The expression of autophagosome marker microtubule-associated protein 1 light chain 3 (LC3) and phosphatidyl-ethanolamine (PE)-modified LC3 (LC3-II) has been shown in the SVZ NPCs and their progeny in the rostral migratory stream and olfactory bulb.^7, 8^

Within the dentate gyrus, the cellular distribution of autophagosomes within NPCs and their progeny remain unknown. Thus, in order to test whether autophagic activity occurs in the NPCs and their progeny as they mature within the SGZ, we utilized the monomeric mCherry-EGFP-LC3 tagged protein that has recently been used to measure autophagic flux within the brain in mature neurons.^10^ Based on the sensitivity of the GFP fluorescent signal, but not mCherry fluorescent signal, to the acidic conditions of the lysosome lumen, this approach allows for detection of autophagosomes (GFP and mCherry expressing puncta (GFP+mCherry+)) and autolysosomes (puncta only express mCherry (GFP−mCherry+).

**Figure 1:**
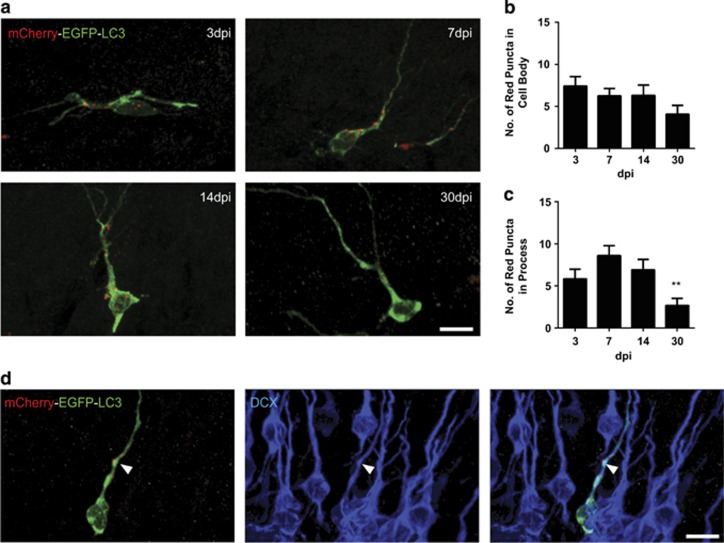
Retroviral labeling with mCherry-EGFP-LC3 shows autophagic activity within NPCs present in the SGZ of the DG. (**a**) Representative images of tandem mCherry-EGFP-LC3 retrovirus-infected cells showing mCherry+ autolysosomes present during the course of NPC maturation at 3, 7, 14 and 30 days post injection (DPI). The autolysosomes were quantified within (**b**) the soma, as well as (**c**) the processes extending from the cell body (*n*=2–3 animals per group, 7–15 cells/animal, one-way ANOVA, **P<0.005 compared with 7DPI). (**d**) Representative image at 7 DPI shows DCX+ cells expressing autolysosomes (arrowhead pointing to autolysosomes present on the dendrite of DCX-expressing neuron). Scale bars=10 *μ*m

We created a mCherry-EGFP-LC3 retrovirus to infect and birthmark the dividing NPCs and examined expression of mCherry-EGFP-LC3 protein at 3, 7, 14 and 30 days post infection (DPI). Infected cells were identified by their pronounced expression of cytoplasmic nonnuclear GFP staining and mCherry-expressing puncta indicative of autolysosomes and suggestive of rapid autophagic flux (Figure 1a). Autolysosomes were clearly visible in the soma and processes of infected cells. At 3–14 DPI, quantification of the number of autolysosomes present in labeled cells revealed no significant difference in the total number of autolysosome in the soma per infected cell (Figure 1b). By 30 DPI there was a significant reduction in number of autolysosomes in the processes, but not within the soma (Figures 1b and c). We further confirmed the infected cells were NPCs and their progeny by identifying that the immature neuronal marker doublecortin (DCX) was expressed in 94±7% and 93±7% of the infected cells at 3 and 7 DPI, respectively (Figure 1d). Together, these findings suggest that autophagy is active in the NPC throughout its development and appears more active in the developing process in the cells that are less than 1-month old.

### Atg5 is required for survival of the progeny of NPCs

**Figure 2:**
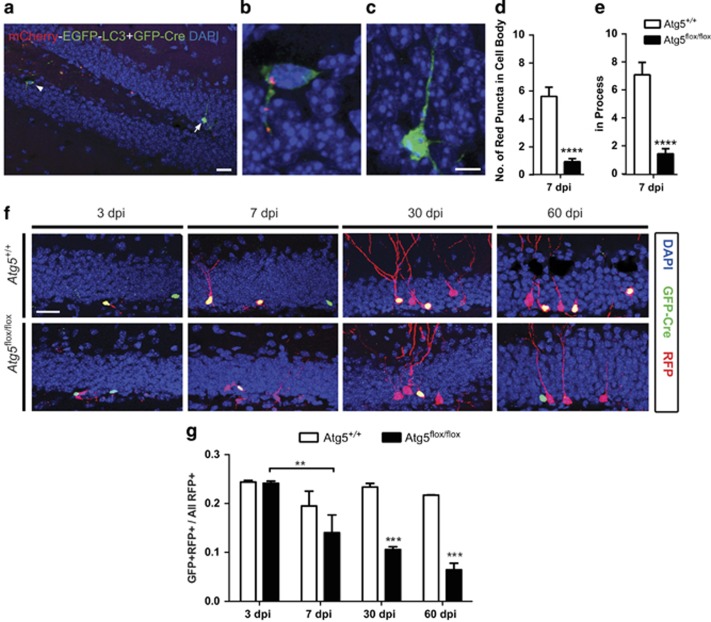
Retrovirus-mediated removal of *Atg5* from dividing NPCs reduces the number of autolysosomes and decreases cell survival. (**a**) Representative image of the dentate gyrus showing two retroviral-infected cells. A cell infected with mCherry-EGFP-LC3 virus is identified by cytoplasmic GFP expression, also identifiable in (**b**) at higher magnification. In contrast, a cell infected with both mCherry-EGFP-LC3 and GFP-Cre is identified by cytoplasmic EGFP and nuclear GFP expression, respectively, also identifiable in (**c**) at higher magnification. Scale bar=20 *μ*m (**a**) and 5 *μ*m (**b** and **c**). The number of autolysosomes in the (**d**) cell body and (**e**) processes of Atg5-null cells in *Atg5*^*flox/flox*^ mice was significantly reduced compared with WT cells in *Atg5*^*+/+*^ mice at 7 DPI (*n*=3 *Atg5*^*+/+*^ mice, *n*=2 *Atg5*^*flox/flox*^ mice; *n*=7–13 cells/animal; cell t(50)=5.7, ****P<0.0001; for processes t(50)=5.0, ****P<0.0001). (**f**) Representative confocal images of GFP-Cre+ (green), RFP+ (red) and double-labeled (yellow; GFP+RFP+) cells at 3, 7, 30 and 60 DPI of retroviruses (*CAG-GFP-Cre* and *CAG-RFP*) into the dentate gyrus of WT (*Atg5*^*+/+*^) and floxed *Atg5* (*Atg5*^*flox/flox*^) mice. Scale bar=20 *μ*m. (**g**) The survival ratio expressed as GFP+RFP+ (yellow) over all RFP+ (red+yellow) cells at 3–60 DPI showing less survival of Atg5-null cells at 30 and 60 DPI (*n*=2–5 animals per group, two-way ANOVA, Bonferroni *post hoc*. **P<0.01, ***P≤0.005)

To determine whether autophagy is essential for the survival of NPCs and their progeny, we used a retrovirus-mediated gene transfer approach to knock out *Atg5* from the dividing NPCs in the adult brain. To determine whether loss of *Atg5* from NPCs would affect autophagic flux, the number of autolysosomes were counted in the *Atg5*^+/+^ and *Atg5*^flox/flox^ mice that were coinjected with the GFP-Cre and mCherry-EGFP-LC3 retroviruses. Cells infected with mCherry-EGFP-LC3 alone expressed GFP in the cytoplasm (Figure 2a arrowhead, higher magnification in Figure 2b), whereas cells infected with both viruses expressed GFP throughout the nucleus and cytoplasm because the GFP expression in GFP-Cre-infected cells is nuclear due to a nuclear localization signal (Figure 2a arrow, higher magnification in Figure 2c). Quantification of the *Atg5-*null and *Atg5*^+/+^-infected cells revealed significantly less autolysosomes in the Atg5-null cells in both the cell body (Figure 2d) and the process (Figure 2e).

In order to fate map and assess the survival of the Atg5-null NPCs, a mixture of retroviral GFP-Cre (*CAG-GFP-Cre*) and control RFP (*CAG-RFP*)^11^ was used to infect the dividing NPCs in the SGZ of the *Atg5*^flox/flox^ mice and wild-type (*Atg5*^+/+^) littermates. This approach was used as it allows for the comparison of the ratio of the Atg5-null infected double-labeled GFP-Cre- and RFP-expressing (GFP+RFP+) cells to all control RFP-expressing (RFP+) cells, as similarly published by our group and others.^11, 12, 13, 14, 15^ As expected in the *Atg5*^+/+^ mice there was no significant difference in survival ratio between 3 and 60 DPI. In contrast, in the *Atg5*^flox/flox^ mice there was a reduction in the survival over time, with *post hoc* analysis revealing a significant reduction in survival between 3 and 7 DPI. The significant reduction in survival of Atg5-null cells at 3 to 7 DPI was associated with a significant reduction in survival ratio between the *Atg5*^flox/flox^ mice and the control *Atg5*^+/+^ mice at 30 and 60 DPI (Figures 2f and g). The reduced survival ratio outcome was also supported by the cell counts for average number of Cre-GFP- and RFP-infected cells that revealed a significant reduction in number of Cre-GFP-infected cells at 7 DPI in the *Atg5*^flox/flox^ mice compared with the control *Atg5*^+/+^ mice. The reduction in number of Atg5-null (GFP+) cells occurred in the absence of any difference in number of control RFP-infected cells at any time point (Supplementary Figure 1). Thus, overall the results suggest that Atg5 is required for survival of progeny of the NPCs during the critical window of 3 to 7 days. These results suggest that the significant reduction in survival of Atg5-null NPCs is mediated by autophagy.

### The progeny of Atg5-null NPCs have a delay in neuronal maturation in the absence of a change in fate

**Figure 3:**
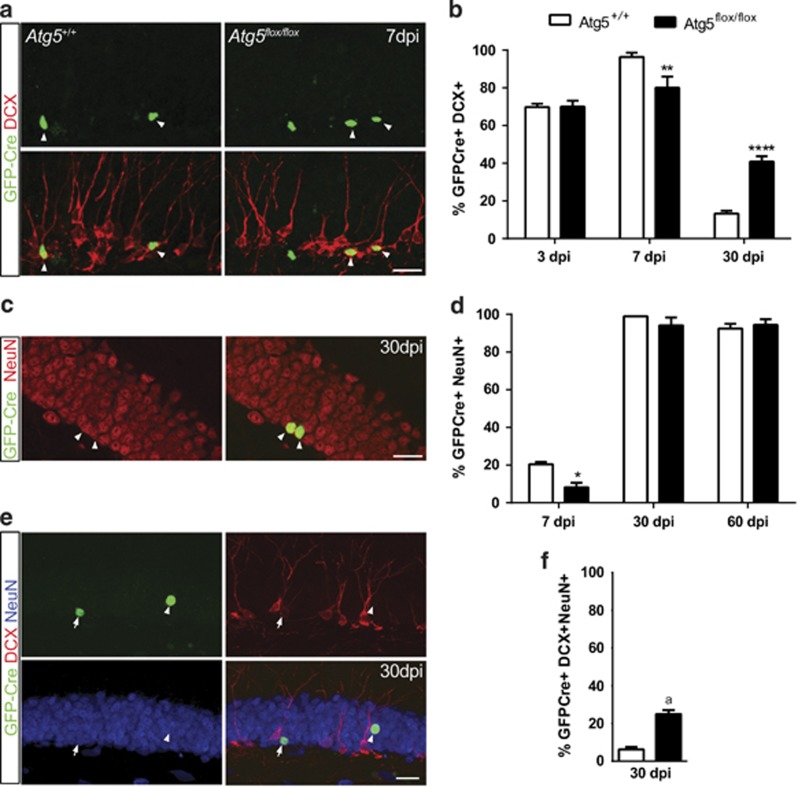
The progeny of the surviving Atg5-null NPCs have a delay in neuronal maturation in the absence of a change in fate. (**a**) Representative image of GFP-Cre+ cells at 7 DPI colabeled with DCX (arrowheads GFP+DCX+ colabeled cells). (**b**) Quantification of GFP-Cre+ expressing DCX showing proportionally less Atg5-null cells express DCX at 7 and more at 30 DPI (*n*=3–5 animals per group, *n*=17–23 cells/animal, two-way ANOVA, Bonferroni *post hoc*, **P<0.01, ****P<0.0001). (**c**) Representative image showing two GFP-Cre+ cells expressing NeuN at 30 DPI. (**d**) Quantification of GFP-Cre+ expressing NeuN showing less Atg5-null cells express NeuN+ cells at 7 DPI (*n*=3–5 animal per group, *n*=17–23 cells/animal, two-way ANOVA, Bonferroni *post hoc*, *P<0.05). (**e**) Representative image of GFP-Cre+ (green) cells expressing DCX (red) and/or NeuN (blue) (arrowhead=GFP-Cre+NeuN+DCX− and arrow=GFP-Cre+NeuN+DCX+). (**f**) Quantification showing more Atg5-null cells coexpressed DCX and NeuN at 30 DPI (*n*=4 *Atg5*^*+/+*^, *n*=5 *Atg5*^*flox/flox*^ mice, *n*=17–23 cells/animal, ^a^t(7)=7.0, *P*=0.0002). Scale bars=20 *μ*m

To examine whether the loss of *Atg5* altered the maturation or fate of the NPCs, we quantified the expression of different cell lineage markers in the Atg5-null cells in the *Atg5*^flox/flox^ mice versus the Atg5-expressing cells in the control *Atg5*^+/+^ mice. At 3 DPI, there was no difference in the proportion of Atg5-null and Atg5-expressing cells that were dividing, as measured by GFP+ cells expressing the cell cycle marker, Ki67 (Supplementary Figure 2). Examination and quantification of the Atg5-null and Atg5-expressing cells that expressed the immature neuronal marker DCX varied significantly between 3 and 30 DPI (Figures 3a and b). At 3 DPI, there was a similar proportion of Atg5-null and Atg5-expressing cells that expressed DCX. At 7 DPI, there was a significant reduction in the proportion of surviving Atg5-null cells that expressed DCX. In contrast, at 30 DPI there was a significant increase in the proportion of surviving Atg5-null cells that expressed DCX. This finding raised the hypothesis that Atg5-null adult-generated cells may have a delay in their neuronal maturation. In support of this hypothesis there was a significant reduction in the proportion of Atg5-null cells at 7 DPI that expressed the mature neuronal marker NeuN (Figures 3c and d). This effect was transient and almost all NPCs expressed NeuN at 30 and 60 DPI. In further support of the Atg5-null cells having a transient delay in maturation, the proportion of 30 DPI Atg5-null cells that expressed both DCX and NeuN was significantly increased (Figures 3e and f). By combining our analysis of the number of surviving Atg5-null and Atg5-expressing cells between 3 and 60 DPI (Figure 2) and average percentage that become neurons at 60 DPI (NeuN+) (Figure 3c), we can further estimate that removal of *Atg5* reduced the number of new neurons by approximately twofold (neuronal survival rate between 3 and 60 days: 53% wild-type (WT) and 27% *Atg5*^fl/fl^). These results therefore support that Atg5 has a pro-survival role and is required for the temporal development of the young immature neuron as it develops into an adult-generated neuron.

### The progeny of Atg5-null NPCs have a transient reduction in spine density

**Figure 4:**
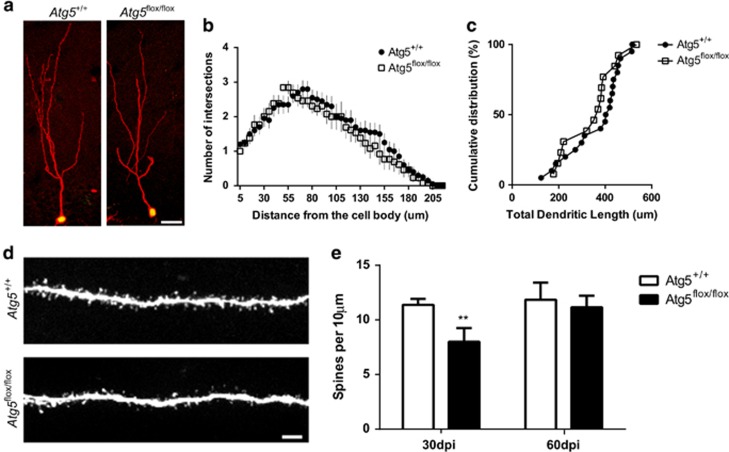
Surviving Atg5-null cells show normal dendritic development but delay in the development of spines. (**a**) Representative confocal images showing WT and Atg5-null neurons (GFP+RFP+) at 30 DPI. Scale bar=20 *μ*m. (**b**) Sholl analysis of dendritic arbors in WT and Atg5-null neurons shows no difference in the complexity of dendrites (*n*=2 animal per group, 13–20 dendrites/animal). (**c**) Cumulative frequency distribution of WT and Atg5-null cells shows no difference in total dendritic length (*n*=2 animal per genotype, 6–13 cells/animal). (**d**) Representative image of spines along a dendritic segment from WT and Atg5-null neurons at 30 DPI. Scale bar=2 *μ*m. (**e**) Quantification of the number of spines per 10 *μ*m reveals a reduction in spine density in Atg5-null neurons at 30 DPI (*n*=2 *Atg5*^*+/+*^, *n*=4 *Atg5*^*flox/flox*^ mice; *n*=3–7 dendrites/animal). **P<0.01

The signification reduction in survival of Atg5-null cells was associated with a delay in neuronal maturation. Given autophagy has been recently shown to regulate embryonic spine pruning^16^ and our observation of autolysosomes present in the dendrites of adult-generated hippocampal cells (Figure 1), we examined whether removal of *Atg5* altered dendritic outgrowth and spine density in adult-generated cells (Figure 4). Sholl analysis and examination of the total dendritic length revealed no differences in the Atg5-null cells compared with Atg5-expressing cells (Figures 4a–c). In contrast, there was a significant reduction in spine density in the Atg5-null *versus* Atg5-expressing cells at 30 DPI (Figures 4d and e). The reduction in spine density was transient and was not significantly different by 60 DPI (Figure 4e). These findings correlate with the delay in neuronal maturation observed by lineage analysis (Figure 3) and further support that *Atg5* is required during the development of the immature neurons.

### Inactivation of Bax restores Atg5-induced deficits in neurogenesis

**Figure 5:**
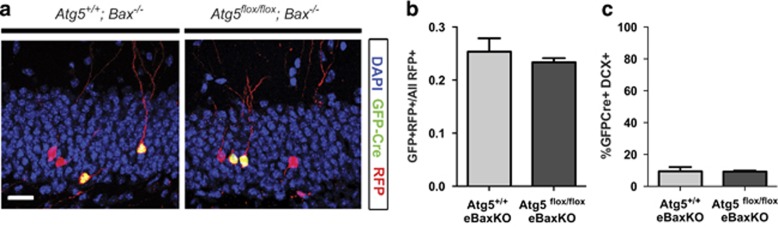
Reduction in survival and maturation of Atg5-null NPCs is Bax-dependent. (**a**) Representative images and (**b**) quantification of 30-day-old retrovirus-infected cells in the *Atg5*^*+/+*^*;eBaxKO* and *Atg5*^*flox/flox*^*;eBaxKO* mice showing no difference in survival ratios at 30DPI (*n*=3 animals/group). (**c**) Quantification of GFP-Cre+ cells showing no difference in proportion of that express DCX at 30 DPI in the *Atg5*^*+/+*^*;eBaxKO* and *Atg5*^*flox/flox*^*;eBaxKO* mice (*n*= 2 animals/group, 20–21 cells/animal). Scale bar=20 *μ*m

The significant reduction in number of Atg5-null cells led us to hypothesize that the Atg5-null cells are dying likely by apoptosis. Indeed, the number of adult-generated neurons is well known to be only a fraction of the number of dividing NPCs because of apoptotic cell death that occurs during neurogenesis.^11, 17, 18, 19^ Therefore, we tested whether blocking apoptosis would prevent the loss of maturing Atg5-null cells. Specifically, we determined whether virally infected Atg5-null cells would have enhanced survival in mice that lacked the essential apoptotic protein, Bax.^20^ Consistent with a Bax-dependent process mediating apoptosis in adult NPCs in development, we have previously reported that embryonic Bax knockout (*Bax*^*−/−*^) mice have a significant increase in average number of virally labeled cells compared with WT control mice, and can rescue maturing NPCs that die due to a lack of Bcl-2.^15^ Therefore, we bred embryonic Bax knockout (*Bax*^*−/−*^) mice with *Atg5*^flox/flox^ mice to create *Atg5*^*+/+*^*Bax*^*−/−*^ and *Atg5*^flox/flox^*Bax*^−/−^ littermate mice and the labeled cells were examined 30 DPI with the dual retroviral labeling strategy. In the absence of *Bax* there was no difference in the survival of Atg5-null or Atg5-expressing retroviral-transduced cells (Figures 5a and b). Similarly, there was no difference in the percentage of the Atg5-null or Atg5-expressing that expressed DCX at 30 DPI (Figure 5c). This is in sharp contrast to the delay in maturation and significant increase in the proportion of surviving Atg5-null cells that expressed DCX at 30 DPI in the presence of Bax (Figure 3a). Together, these findings support that removal of Bax can rescue both the loss of the Atg5-null cells and their delay in maturation, supporting that Atg5 functions in the maturing NPCs upstream of the Bax apoptotic pathway.

### Atg5 is required for exercise-induced increase in survival of NPCs

**Figure 6:**
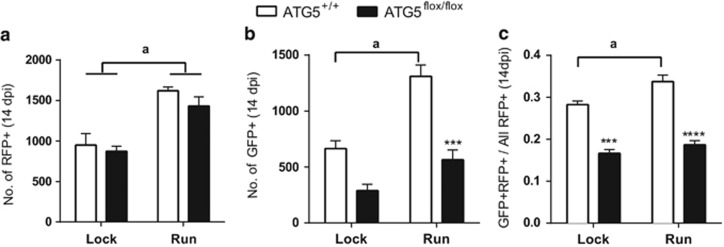
Atg5 is required for exercise-induced increase in developing NPCs. (**a**) Mice with free access to running wheel compared with a locked wheel showed an increase in the number of non-Cre-infected (RFP^+^) cells in both the *Atg*5^flox/flox^ and WT mice. (**b**) Numbers of GFP-Cre+ cells were increased in the *Atg5*^*+/+*^ run *versus Atg5*^*+/+*^ lock mice and comparable in *Atg5*^*flox/flox*^ run *versus Atg5*^*flox/flox*^lock. (**c**) The survival ratio is higher in the *Atg5*^*+/+*^ run *versus Atg5*^*+/+*^ lock mice and comparable in *Atg5*^*flox/flox*^ run *versus Atg5*^*flox/flox*^lock (for **a**–**c**, *n*=4 *Atg5*^*+/+*^ lock, *n*=3 *Atg5*^*flox/flox*^ lock, *n*=4 *Atg5*^*+/+*^ run, *n*=6 *Atg5*^*flox/flox*^ run). Bonferroni *post hoc*, ***P≤0.0002, ****P<0.0001, ^a^significant difference when compared with lock group of same genotype

Running has been shown to increase autophagy within the brain,^21^ and therefore we tested whether Atg5 was required for exercise-induced increases in neurogenesis. As expected, the *Atg5*^flox/flox^ and the WT (*Atg5*^+/+^) mice that had free access to a running wheel had an increase in the number of control non-Cre infected (RFP^+^) cells compared with mice that had access to a locked wheel (Figure 6a). Examination of the WT mice revealed that there was significant increase in Cre-infected (GFP+) cells in mice given access to the running wheel (Figure 6b). Analysis of the survival ratio further showed in the WT mice a significant increase in survival ratio (Figure 6c). In contrast, the *Atg5*^flox/flox^ mice that were exposed to either a locked or running wheel had significantly less Atg5-null (GFP+) cells compared with WT mice (Figure 6b). In addition, the *Atg5*^flox/flox^ mice compared with WT mice had a significant reduction in the survival ratio in the locked or free running wheels (Figure 6c). These changes occurred in the context of similar distances ran between the WT and *Atg5*^flox/flox^ mice (WT 210 836±32 164 m *versus Atg*5^fl/fl^ 159 909±27 434 m). Therefore, overall these results suggest that running cannot rescue the Atg5-null survival phenotype, with Atg5 being required for running-induced increases in NPCs.

## Discussion

Here we demonstrate that autophagy is active in the progeny of the NPCs and that Atg5 is required for survival of the progeny of the adult NPCs. Autophagic flux is present in the progeny of the hippocampal NPCs, with autolysosomes being detected within the soma and dendrites. Functionally, retroviral infection of Cre in *Atg*5^flox/flox^ mice reduces autolysosomes in Atg5-null cells and is associated with a significant loss of the survival of the progeny of the NPCs during the first 7 days of their maturation. The reduction in survival is accompanied by a delay in the maturation of the adult-generated neuron including a transient reduction in spine density in the absence of altering proliferation or neuronal fate. The *in vivo* complementation experiments with *Bax* knockout mice indicate that the reduction in Atg5-null immature neurons occurs upstream of a Bax-mediated death. Moreover, running, which is a known inducer of autophagy, was insufficient to reverse the significant survival phenotype of the *Atg5-*null NPCs.

These findings suggest that Atg5 is one of the gatekeepers that controls which NPCs in the adult brain survive during their development into new functional neurons.

These findings add to the recent work of others that have examined autophagy and adult neurogenesis *in vivo*^7, 8, 9^ and show for the first time that autophagy has a pro-survival role during the development of adult-generated hippocampal neurons. Our conclusions further provide evidence that 3–7-day-old cells undergo a critical period for survival that requires Atg5. We also show the pro-survival effect of Atg5 in the NPCs can occur independent of effects in the embryonic or early postnatal brains, independent of altering cell proliferation, as well as independent of effects in the adult stem cell population as this retrovirus targets dividing NPCs. To delineate whether Atg5 is required in the stem cells, future studies will require an inducible system to target the slowly dividing stem cells.

Removal of Atg5 in the NPCs also resulted in a significant delay in their maturation. The timing of the delay in maturation was observed between 1 week and 1 month post viral infection. During this time it is well known that there is a requirement of neuronal activity to promote cell survival through activity-dependent mechanisms, including the NPCs having a lower threshold for long-term potentiation.^3, 22, 23, 24^ Therefore, the delay in maturation may contribute to the reduction in the survival of Atg5-null cells. This is an exciting possibility as a variety of studies have identified that neuronal activity can regulate autophagy through a variety of mechanisms, such as activity-dependent retrograde transport of autophagosomes and presynaptic AMPA receptor degradation.^25, 26, 27, 28^ Together, these findings lead to the hypothesis that autophagy and neuronal activity may coregulate the survival of the maturing NPCs.

The reduction in survival and delay in maturation of Atg5-null cells occurs independent of affecting the differentiation of the dividing NPCs into mature dentate neurons. Almost all of the Atg5-null NPCs that survive develop into NeuN-expressing neurons within 2 months. This is surprising as *in vivo* studies examining autophagy and neurogenesis support that at least Ambra1, Beclin1 and FIP200 are required for neuronal differentiation in the progeny of adult NPCs.^7, 8^ There are many possible explanations that could account for this difference. One hypothesis is that the retrovirus-transduced dividing NPCs could be past the stage of neuronal lineage commitment. This explanation however seems unlikely as a decrease in neuronal lineage in retroviral-transduced NPCs was recently published.^29^ A more likely hypothesis may be that the requirement of autophagy for differentiation may be different within the embryo *versus* adult NPCs and their progeny. This could arise from intrinsic differences in the embryonic or adult cell populations and in compensation that occurs in the embryonic *versus* adult neurogenic niches. This is supported by the variability in phenotypic outcomes reported in the *Atg5* ablation *in vivo* mouse models. Conditional removal of *Atg5* at E10.5 within the nestin-expressing stem and NPCs induced neurodegeneration and intracellular protein accumulation, in the absence of gross differences in differentiation.^30^ More recently, inducible knockdown of Atg5 or overexpression of Atg5 in the cortical NPCs at E13 suggested Atg5 was essential to promote neuronal lineage commitment.^9^ Using the same methodology at E16, this group also recently reported that Atg5 promotes astrocyte differentiation within the cortex.^31^ In contrast, we find removal of Atg5 does not alter the fate of the dividing NPC progeny.

Our study also demonstrates that removal of Bax can rescue the loss of the Atg5-null cells, supporting that Atg5 functions upstream of the Bax apoptotic pathway in the immature neurons. Despite autophagy and apoptosis being distinct cellular processes, the protein networks that control their regulation and execution are highly interconnected.^32^ For example, Atg5 can induce apoptosis through cleavage of the nonconjugated form of Atg5 by calpains that then directly interacts with the Bcl-2 family member, B-cell lymphoma-extra larger (Bcl-xL).^33^ Although both Atg5 and truncated Atg5 do not directly bind Bax, it remains unknown whether the truncated Atg5 can promote apoptosis through activation of Bax–Bak-like molecules by the inactivation of Bcl-xL. As truncated Atg5 promotes apoptosis and we have found deletion of Atg5 promotes the death of the NPC progeny, it is unlikely that truncated Atg5 is involved in the death of the Atg5-null cells. This is also supported by our recent finding that calpain-1 and calpain-2, the proteases that cleave Atg5, have no deficits in adult neurogenesis.^34^

Together, these data suggest Atg5 may be working through the canonical autophagy pathway to regulate the survival of the developing NPCs. If this is true, then inducing autophagy should be able to rescue the survival phenotype. This could happen through an Atg5-dependent conventional or Atg5-independent pathway.^35^ We show that running requires Atg5 in the NPCs to increase the survival of NPCs and thus cannot rescue the Atg5-null survival phenotype. Further studies will be required to elucidate in more detail the role of autophagy in exercise-induced neurogenesis in the brain. For example, this study examined voluntary running, leaving it unknown if our findings would generalize to all forms of exercise. In addition, to directly test the hypothesis that the Atg5-null survival deficits that occur in naive and running mice are Atg5 dependent, an inducible NPCs mouse model is required to elucidate whether a gain-in-function experiment can rescue the survival deficits of the Atg5-null cells.

In conclusion, our findings support a cell-autonomous requirement for Atg5 in adult immature neurons. This finding provides new information about the regulation of survival of the NPC progeny that is important for designing therapeutic strategies aimed at promoting endogenous repair and improving the success of neural transplants. Our findings also have additional implications because of exponential growth of the development of therapeutics that inhibit and induce autophagy as an intervention for various types of human disorders.^36^ For example, the autophagic inhibitors that are being tested as cancer therapeutics may have unexpected deleterious side effects of reducing neurogenesis. However, on the converse, the autophagic inducers that are being designed for neurodegenerative diseases could exert additional beneficial effects through promotion of adult neurogenesis.

## Materials and Methods

### Animals

The C57BL/6 mice were purchased from Charles River (Saint-Constant, QC, Canada, male, 7–12 weeks old). The mouse lines that were genotyped according to previously published protocols included the: floxed Atg5 mice from RIKEN^30^ and Bax knockout mice from Jackson Laboratories (Bar Harbor, ME, USA).^37^ A range of 2–9 mice were included in each experimental group. Animal procedures were performed within the guidelines of the Canadian Council on Animal Care and were approved by the University of Ottawa Animal Care Committee.

Mice were housed in a 12-h light/dark cycle with free access to food and water. For exercise experiment (Figure 6), mice were singly housed with free access to a low-profile wireless running wheel or a locked wheel (Med Associates, St. Albans, VT, USA) for 1 week before and 2 weeks post retroviral infection.

### Retrovirus and stereotaxic injection

The retroviral vectors *CAG-GFP-Cre* and *CAG-RFP* and the corresponding packing and envelope constructs were generously provided by Dr. Fred Gage (The Salk Institute, La Jolla, CA, USA). The retroviruses were generated as previously described.^11^ The titers of *GFP-Cre* and *RFP* viruses were determined by live tittering using 293T cells and ranged between 2 and 4 × 10^8^ infectious units (IU) per ml. The *GFP-Cre* and *RFP* virus was injected in a 1 : 1 ratio (volume 1.5 *μ*l), except for mice used for phenotyping that were injected with only GFP-*Cre* (volume 1 *μ*l). The mCherry-EGFP-LC3B plasmid (Addgene, Cambridge, MA, USA, 22418) was used to produce retroviruses as described above and 1.5 *μ*l was injected into the dentate. In order to target the SGZ, all retroviruses were bilaterally injected into the dentate gyrus (−1.7 mm anterior/posterior, ±1.2 mediolateral, −2.4 mm dorsoventral from Bregma) of mice (7–9 weeks old) during stereotaxic surgery. mCherry-EGFP-LC3B retrovirus was also coinjected with the GFP-Cre retrovirus in both Atg5^+/+^ and Atg5^flox/flox^ mice to determine the effect of Atg5KO on autophagic puncta. Infected cells were distinguished based on either nuclear GFP expression (GFP-Cre infected), cytoplasmic GFP expression (mCherry-EGFP-LC3B infected) or both nuclear and cytoplasmic GFP (GFP-Cre and mCherry-EGFP-LC3B dual-infected) cells.

### Tissue processing and immunohistochemistry

Mice were anesthetized and transcardially perfused with cold phosphate-buffered saline (PBS, pH7.4) followed by 4% paraformaldehyde in PBS. Brains were removed and postfixed for 1 h in 4% paraformaldehyde and then transferred into 30% sucrose with 0.1% sodium azide in PBS. Brains were sectioned coronally into 30 *μ*m slices on a freezing microtome and stored in PBS with 0.1% sodium azide. Histology was performed using free-floating methodology with sections being washed in PBS and incubated in 0.1% Tween-20 and 0.1% Triton X-100 in PBS with corresponding primary antibodies at 4 °C overnight. The following primary antibodies were used: chicken anti-GFP (GFP-1020, Aves, Labs, Inc., Tigard, OR, USA, 1 : 5000), rabbit anti-dsRed (632496, Clontech, Laboratories, Inc., Mountain View, CA, USA, 1 : 5000), rabbit anti-Ki67 (275R-14, Cell Marque, Rocklin, CA, USA, 1 : 100), goat anti-DCX (sc-8066, Santa Cruz, Biotechnology, Inc., Dallas, TX, USA, 1 : 500) and mouse anti-NeuN (MAB377, Millipore, Etobicoke, ON, Canada, 1 : 500). On day 2, sections were washed in PBS, incubated with corresponding Cy2-, Cy3- and Cy5-conjugated IgG antibodies (Jackson ImmunoResearch, Laboratories, Inc., West Grove, PA, USA, 1 : 500) for 1 h at room temperature, stained using 4',6-diamidino-2-phenylindole (DAPI, 10236276001, Roche, Laval, QC, Canada, 1 : 10 000, 3 min) and washed in PBS.

Cells in the SGZ were counted using an Olympus BX51 fluorescent microscope (Olympus Canada Inc., Richmond Hill, ON, Canada) using unbiased histological approaches in every ninth serial section throughout the dentate, as previously described.^38^ The quantification of colabeled cells was performed using a Zeiss LSM510-META confocal microscope (Carl Zeiss, North York, ON, Canada), as previously described.^38^ For mCherry-EGFP-LC3 puncta counts, the images were acquired at the confocal microscope using 63 × objective and all puncta were counted irrespective of their size within the infected cells. For spine analysis, dual-labeled cells were imaged at × 63 (oil immersion) with a Quorum Spinning-disk confocal microscope (Quorum Technologies Inc., Guelph, ON, Canada) at emission wavelengths of 406, 490 and 561. MetaMorph automation and image acquisition software (Molecular Devices, Sunnyvale, CA, USA) was used to create a high-resolution three-dimensional representation of spines throughout the visible dendritic arbor using 0.5 *μ*m *Z*-plane optical sectioning in combination with a tile-scan module. Images were subsequently stitched and flattened in MetaMorph and exported to NeuronStudio (CNIC, Ichan School of Medicine at Mount Sinai, New York, NY, USA) to measure neurite length. Spines were manually quantified from a single neurite that spanned the hippocampal molecular layer (top of the granule cell layer to the hippocampal fissure) per cell in Fiji image processing software (ImageJ, National Institutes of Health (NIH), Bethesda, MD, USA). Spine density (spines/10 *μ*m) was calculated as the quotient of the number of spines over neurite length multiplied by 10. Although not specifically quantified, spine density was observed to be similar for any individual cell throughout the molecular layer.

### Statistics

All data are reported as mean±S.E.M. and statistical analysis performed using GraphPad Prism (v6.0) software (GraphPad Sofware, Inc., La Jolla, CA, USA). Experiments with two groups were analyzed by the two-tailed Student's *t-*test. Analyses of three or more groups were performed using an ANOVA test followed by Bonferroni *post hoc*. Statistical significance was defined as *P*<0.05.

## Abbreviations

Atg5: autophagy related gene 5
Bax: Bcl-2-associated X protein
NPC: neural progenitor cell
SVZ: subventricular zone
SGZ: subgranular zone
LC3: microtubule-associated protein 1 light chain 3
DCX: doublecortin
