# Supplementary figures and images for: Knockout of Atg5 delays the maturation and reduces the survival of adult-generated neurons in the hippocampus
Source: Cell Death Dis. 2016 Mar 3;7(3):e2127–. doi: 10.1038/cddis.2015.406 (PMC4823925; doi:10.1038/cddis.2015.406)

Supplemental Figure 1

**a**

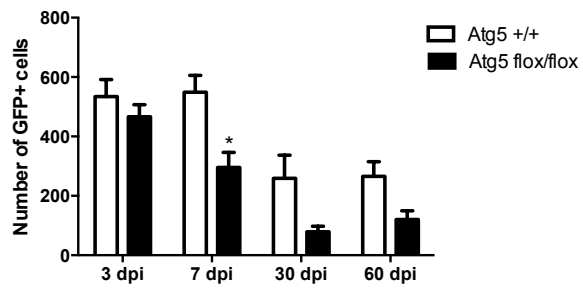

**b**

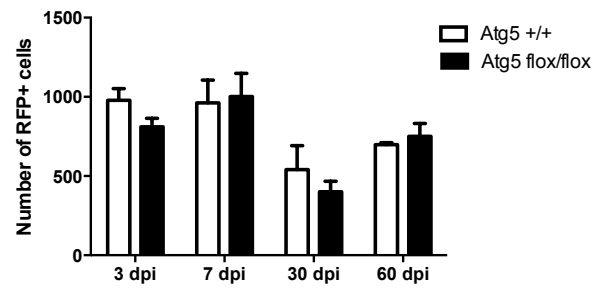

Supplement: Supplementary Figure 1 [file cddis2015406x1.pdf]

**a**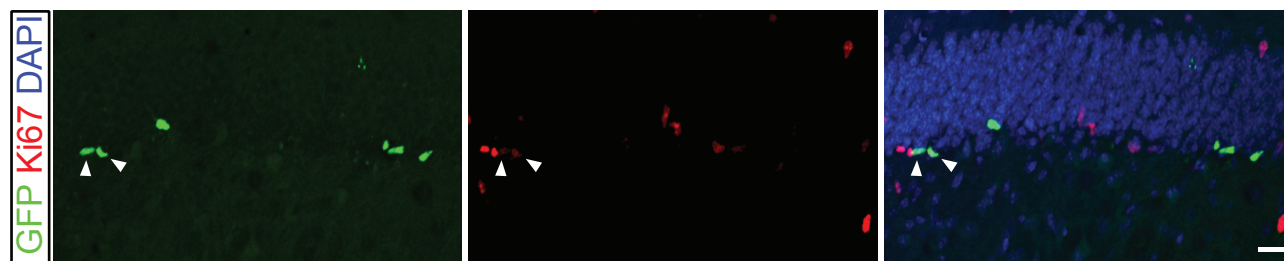**b**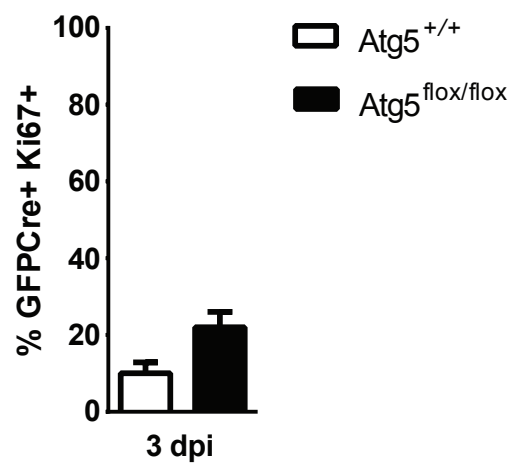

Supplement: Supplementary Figure 2 [file cddis2015406x2.pdf]
