# Supplementary material for: Knockout of Atg5 delays the maturation and reduces the survival of adult-generated neurons in the hippocampus
Source: Cell Death Dis. 2016 Mar 3;7(3):e2127–. doi: 10.1038/cddis.2015.406 (PMC4823925; doi:10.1038/cddis.2015.406)
Supplement: Supplementary Figure Legends [file cddis2015406x3.doc]

**Supplemental Figure 1** **Retrovirus-mediated removal of *Atg5* from dividing NPCs.**

**(a)** Average number of GFP-Cre+ (green) and **(b)** control RFP+ (red) cells at 3, 7, 30 and 60 dpi of retroviruses (*CAG-GFP-Cre* and *CAG-RFP*) into the dentate gyrus of wild-type (WT, *Atg5^+/+^*) and floxed *Atg5* (*Atg5^flox/flox^*) mice. (n=2-5 animal per group, Two-way ANOVA, Bonferroni posthoc * p<0.05 *Atg5^flox/flox^ versus Atg5^+/+^* at 7 dpi).

**Supplemental Figure 2** **Retrovirus-mediated removal of *Atg5* from dividing NPCs does not alter proportion of NPCs in cell division.**

**(a)** Representative confocal images of GFP-Cre+ (green), Ki67+ (red) and double-labeled (arrowheads yellow; GFP+Ki67+) cells in the dentate gyrus of a floxed *Atg5* (*Atg5^flox/flox^*) mice (blue=DAPI nuclear counterstain) scale bar=20um **(B)** Percentage of GFP-Cre+ cells that are labeled with Ki67 shows no difference in percentage of dividing cells between wild-type (WT, *Atg5^+/+^*) and floxed *Atg5* (*Atg5^flox/flox^*) mice. t-test, t(6)=2.1, p>0.05
